# Complete Genome Annotation of Mycobacteriophage Kremtemulon

**DOI:** 10.17912/micropub.biology.001985

**Published:** 2026-03-06

**Authors:** Cole R. Jirsa, Bethany M. Wise, Kaylia Edwards, Jerusalem Mussie, Owen Tolbert, Danielle M. Heller

**Affiliations:** 1 Department of Science Education, Howard Hughes Medical Institute, Chevy Chase, Maryland, United States; 2 University of Maryland, Baltimore County, Baltimore, Maryland, United States

## Abstract

Kremtemulon is a siphovirus isolated on the host bacterium *Mycobacterium smegmatis* mc ^2^ 155 with a genome spanning 51,438 bp in length. Kremtemulon encodes 87 putative genes, 36 of which have predicted functions, and based on gene content, is assigned to Cluster A and Subcluster A4. Kremtemulon forms turbid plaques and encodes for both an immunity repressor and a tyrosine integrase, suggesting that Kremtemulon is a temperate phage.

**Figure 1. Morphology and genome of mycobacteriophage Kremtemulon:**
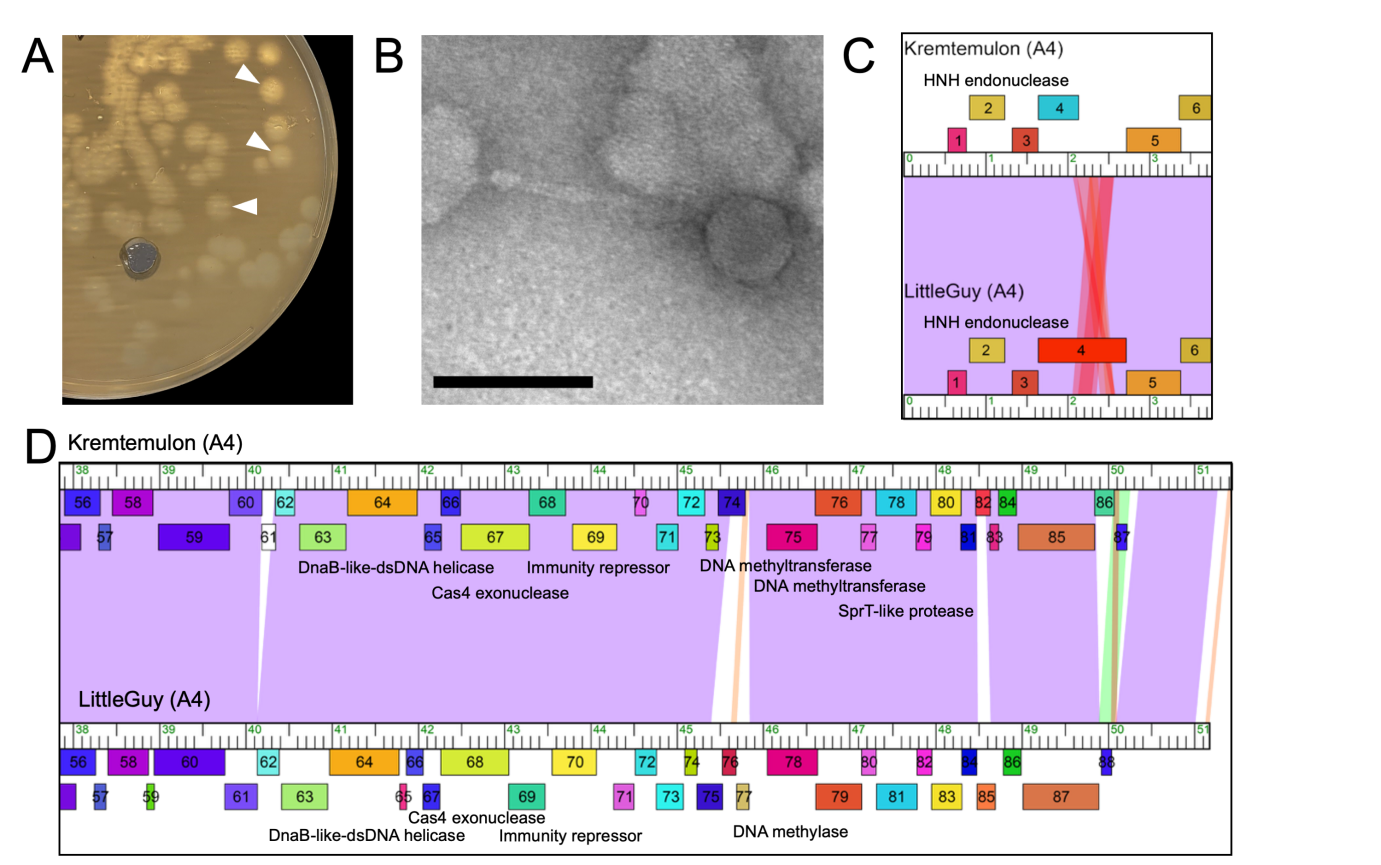
(A)&nbsp; Plaque assay showing large, turbid plaques formed by Kremtemulon on *Mycobacterium smegmatis* mc²155. White arrows highlight representative plaques to help distinguish from the hole in the bottom-left of the top agar lawn. (B)&nbsp; Transmission electron micrograph (TEM) of Kremtemulon. Kremtemulon is a siphovirus with an icosahedral head 57 nm in diameter and a long, flexible, non-contractile tail 136 nm in length. The scale bar represents 100 nm. (C and D) Genome alignment of two genome regions from cluster A4 phages Kremtemulon and LittleGuy. The ruler represents the genome coordinates in 1 kbp units with boxes above and below the ruler representing genes transcribed in the forward and reverse orientations, respectively. Kremtemulon gene *4* (C) is truncated, relative to LittleGuy gene *4* . There are multiple indels in the right arm (D) of the Kremtemulon genome relative to LittleGuy. Of note, Kremtemulon encodes orphan gene *61* and lacks homologs of LittleGuy genes *76* and *77* , which are conserved in other A4 genomes, indicating a likely deletion event. Kremtemulon gene *86* is not conserved in LittleGuy and represents a weakly conserved gene found in only a subset of A4 phages.

## Description

Bacteriophages are critical components for understanding microbial ecology and offer promising alternatives to traditional antibiotics (Hatfull, 2020). Here, we report the isolation and characterization of Kremtemulon, a mycobacteriophage that infects *Mycobacterium smegmatis* mc²155.

Kremtemulon was isolated from a soil sample collected near a tranquil stream bank in Williamsburg, VA (GPS coordinates: 37.271102º N, 76.716221º W). After collection, the soil sample was resuspended in liquid medium (Middlebrook 7H9 broth supplemented with 145 mM NaCl, 5% Albumin fraction V, 2% dextrose, and 1 mM CaCl _2_ ) and the suspension filtered (0.22-μm pore size). The filtrate was enriched for mycobacteriophages by inoculation with *M. smegmatis* mc²155 followed by incubation at 37 ºC with constant agitation for 48 h. The enriched sample was re-filtered and the filtrate plated with Middlebrook 7H9 top agar supplemented with 1 mM CaCl _2_ and *M. smegmatis* mc ^2^ 155 to isolate plaques, which were then subjected to multiple rounds of plaque purification on similar top agar lawns. Plates with a high density of plaques were flooded with phage buffer (10 mM Tris pH 7.5, 10 mM MgSO _4_ , 68 mM NaCl, 1 mM CaCl _2_ ), and the collected liquid was filtered to generate a high-titer lysate. Kremtemulon forms large, turbid plaques, which is indicative of a temperate phage ( Figure 1A ). Transmission electron microscopy with negative staining (1% uranyl acetate) revealed that Kremtemulon is a siphovirus ( Figure 1B ), with an icosahedral head measuring 57 ± 1.0 nm in diameter (mean ± standard error, *n* =10 viral particles) and a non-contractile tail measuring 146 ± 4.8 nm in length (mean ± standard error, *n* =8 viral particles).

Kremtemulon genomic DNA was isolated from a high titer lysate using the Promega Wizard DNA Clean-Up Kit. Sequencing of Kremtemulon's genome was performed using an Illumina MiSeq platform (v3 reagents) at the Pittsburgh Bacteriophage Institute, with a library prepared using the NEBNext Ultra II FS DNA Library Prep Kit. This produced 557,541 150-base single-end reads resulting in ~1,534x coverage of the genome. Assembly of the raw reads was completed using Newbler v2.9 (Silva et al., 2013)&nbsp;, and assembly quality and completeness were confirmed with Consed v29 (Gordon & Green, 2013)&nbsp; using default parameters. The Kremtemulon genome is 51,438 base pairs in length with a GC content of 63.9%, similar to the 67.4% GC content of it its isolation host *M. smegmatis* mc ^2^ 155. The Kremtemulon genome contains 3′ single-stranded overhanging ends (5′-CGGCCGGTAA).

Annotation of the Kremtemulon genome was performed using the annotation platforms DNAMaster (http://cobamide2.bio.pitt.edu/) and PECAAN (v20240320; https://discover.kbrinsgd.org/). Open reading frames were first predicted using Glimmer v3.02 (Delcher et al., 2007)&nbsp; and GeneMark v4.28 (Besemer & Borodovsky, 2005), and then manually refined using the additional tools Starterator v558 for start site determination and Phamerator v3 (Cresawn et al., 2011)&nbsp;for comparative genomic visualization. Functional assignments were informed by BLASTp v2.13.0 (Altschul et al., 1990)&nbsp;against the NCBI non-redundant and Actinobacteriophage databases (v581), and by HHPred v3 (Söding et al., 2005)&nbsp;searches against the PDB_mmCIF70, Pfam-A v37, SCOPe v2.08, and NCBI Conserved Domain Database v3.20. All software was run using default parameters.

Kremtemulon was assigned to Cluster A and further sorted into Subcluster A4 based on gene content similarity of 35% or higher to other phages in the Actinobacteriophage database (Gauthier & Hatfull, 2023; Russell & Hatfull, 2016). A pairwise comparison between Kremtemulon and LittleGuy reveals 90.29% gene content similarity in these two A4 mycobacteriophages (Russell & Hatfull, 2016). Kremtemulon encodes for a serine integrase (gene *33* ) and a putative immunity repressor (gene *69* ), suggesting that it like many other Cluster A phages may be temperate (Hatfull, 2020; Mavrich & Hatfull, 2019), though the ability of Kremtemulon to form stable lysogens has not yet been experimentally demonstrated.

The Kremtemulon genome does exhibit some features that are distinctive from other cluster A4 mycobacteriophages. Most notably, between genes *74* and *75,* Kremtemulon lacks two conserved genes of unknown function that are typically found near the immunity repressor region in other A4 genomes (e.g., LittleGuy genes *76* and *77* ), suggesting a potential gene deletion event. Additionally, gene *4* is truncated compared to homologous genes in other A4 phages due to a single base change that introduces an early stop codon, resulting in a gene product that is only 163 amino acids as opposed to the 357 amino acid homolog LittleGuy gene *4* . This gene product is annotated as a minor tail protein in many A4 genomes, and the truncation seen in Kremtemulon suggests that the full-length protein is likely not necessary for phage infection or virion assembly. Kremtemulon also contains two genes of unknown function that are uncommon in the Actinobacteriophage database. Gene *86* is uncommon within A4 mycobacteriophage genomes, with homologs found in only two other A4 phages, whereas gene *61* is an orphan gene with no homologous gene sequences identified in any other genomes currently in the Actinobacteriophage database. Altogether, these differences between closely related mycobacteriophage genomes underscore the complex, ongoing evolution of this population.

**Data Accesibility**

Kremtemulon is available on GenBank with the accession number PQ559668 , and the Sequence Read Archive (SRA) number SRX31207614 .
